# Lipophilicity Examination of Some ACE inhibitors andHydrochlorothiazide on Cellulose in RP Thin-Layer Chromatography

**Published:** 2012

**Authors:** Jadranka Odović, Katarina Karljiković-Rajić, Jasna Trbojević-Stanković, Biljana Stojimirović, Sote Vladimirov

**Affiliations:** a *Faculty of Pharmacy, University of Belgrade, Belgrade, Serbia.*; b *Dr Dragiša Mišović Clinical Center, Belgrade, Serbia.*; c *Institute of Urology and Nephrology, Clinical Center of Serbia and School of Medicine, Belgrade, Serbia.*

**Keywords:** Lipophilicity, ACE inhibitors, Hydrochlorothiazide, Thin-Layer Chromatography

## Abstract

In this assay, the evaluation of lipophilicity of four ACE-inhibitors and hydrochlorothiazide (HCTZ) with RP-TLC on cellulose layers was described using three binary solvent systems. The selected ACE inhibitors had sufficiently different structures which can indicate the method suitability for their lipophilicity evaluation as the model substances in comparison with HCTZ.

In addition, the linear relationship between the R_M_-values and composition of mobile phases was established in the current study. From the regression data of the plots, the hydrophobicity parameters, R^0^_M_ and m, were determined and C_0_ parameter was calculated. The correlations between the experimentally obtained hydrophobicity parameters and calculated log *p *values were studied. Furthermore, the obtained results were compared with those previously obtained on RP-18 modified silica gel. Very good correlation (r = 0.91; water-ethanol solvent system) between the chromatographically obtained hydrophobicity parameters and calculated log *p *values confirmed the selection of ACE inhibitors since lisinopril and quinapril were on the opposite sites of linear relationship. The results indicate that cellulose as an easily available sorbent can be successfully used for the lipophilicity investigation of examined substances with RP-TLC.

## Introduction

Lipophilicity is a physicochemical property that has attracted considerable interests in medicinal chemistry, pharmacokinetic and environmental sciences. The important role of lipophilicity in drug research is a consequence of hydrophobic interactions of the drugs with biological targets, penetration across biological membranes during drug transport, as well as toxic aspects of drug action (1, 2).

Lipophilicity is usually characterized through the *n*-octanol/water partition coefficient (log *P*_O/W_). A traditional approach for the determination of lipophilicity of a molecule, *i.e*., of the log *p *value, is the so-called shake flask method (3, 4).

Nowadays, chromatography is known as a unique method which can yield a great amount of quantitatively comparable, precise and reproducible retention data for large sets of structurally different compounds which can be correlated with their physicochemical and biological properties (2).

A separation technique, such as RP-HPLC, is an efficient technique since the measured retention values can be correlated with hydrophobicity parameter log *p *(5-7). Furthermore, there are numbers of studies focused on the investigation and systematic determination of drugs lipophilicity, using the thin-layer chromatography methods, primarily reversed-phase (RP) (8-11), and also normal-phase (NP) TLC (12) chromatography, as well as comparation between HPLC and TLC (13, 14).

The cellulose is a sorbent frequently applied in TLC (usually used in NP-TLC). Considering that in RP-TLC, the stationary phase has to be less polar than the mobile phase it possible be to use the cellulose layers in RP-TLC with suitable selection of mobile phases. The lipophilicity of s-triazine derivatives (15) as well as that of some 3,5-dinitro-benzoic-acid esters (16) were investigated under the condition of RP-TLC on cellulose layers without any impregnation.

Angiotensin-converting enzyme (ACE) inhibitors are used primarily (in some cases as the first choice drugs) for the treatment of hypertension and congestive heart failure. ACE inhibitors are esterified prodrugs. Following the administration, they undergo the hydrolysis into the active diacid metabolites, which exhibit the inhibitory effect on the angiotensin-converting enzyme (17). In pharmaceutical formulations, they are often combined with diuretic-hydrochlorothiazide (HCTZ), to enhance their antihypertensive effect (EnaHEXAL comp. and Lisinopril Sandoz–Sandoz Pharmaceutical; Inhibace plus–Roche).

According to the available literature, a number of authors investigated the relationship between the lipophilicity and the activity of ACE inhibitors (18-22). There are only few papers with systematic investigations of ACE inhibitors lipophilicity (23-25).

**Figure 1:**
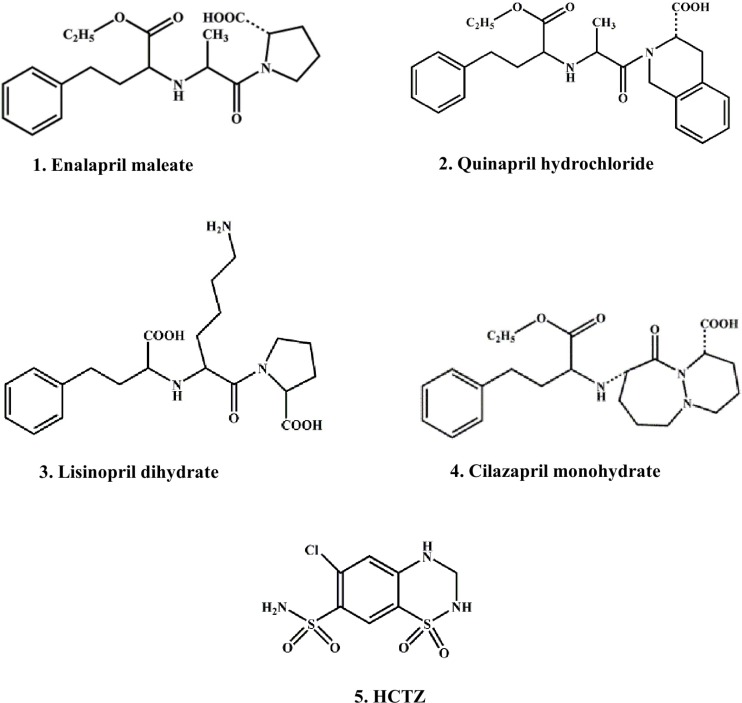
The chemical structures of the investigated drugs

In continuation of our previous researches on chromatographic behavior of ACE inhibitors, under different conditions of TLC (26-28), the aim of this study was the examination of lipophilicity of several ACE inhibitors as well as HCTZ (Figure 1) on cellulose support, as a possible alternative to RP-18 silica gel plates. The selected ACE inhibitors have a sufficiently different structure that can indicate the method suitability for their lipophilicity evaluation as model substances in comparation with HCTZ.

 The certain functional groups significantly affect the polarity of the molecules. As expected, the amino-acid moiety in lisinopril increases its hydrophilic character as well as sulfonamide group in HCTZ. Contrary to these, less polar groups such as esters in enalapril, quinapril and Cilazapril contribute to the increase of their lipophilicity.

In order to evaluate the possible application of cellulose in RP-TLC, the correlation of hydrophobicity parameter, C_0_, obtained on cellulose and RP-18 silica gel plates will be established.

Furthermore, the comparison of chromatographic hydrophobicity parameters for investigated substances, with different log *p *values, could confirm that selected ACE inhibitors and HCTZ can be regarded as the same group under proposed chromatographic conditions.

## Experimental

The three, water-organic modifier, binary solvent systems were used with different volume fractions. All components of mobile phases were of analytical grade of purity.

The investigated substances included: four ACE inhibitor drugs as follows: 1. Enalapril maleate, (S)-1-[N-[1-(ethoxycarbonyl)-3-phenylpropyl]-L-alanyl]-L-proline; 2. Quinapril hydrochloride, [3S-[2[R* (R*)],3R*]]-2-[2-[[1-(ethoxycarbonyl)-3-phenylpropyl] amino]-1-oxopropyl]-1,2,3,4-tetrahydro-3-isoquinolinecarboxylic acid; 3. Lisinopril dihydrate, (S)-1-[N^2^-(1-carboxy-3-phenylpropyl)-L-lysyl]-L-proline dihydrate; 4. cilazapril monohydrate, [1S-[1*α*,9*α *(R*)]]-9-[[1-(ethoxycarbonyl)-3-phenylpropyl] amino] octahydro-10-oxo-6H-pyridazino [1,2-a] [1,2] diazepine-1-carboxylic acid monohydrate and HCTZ, 6-chloro-3,4-dihydro-2H-1,2,4-benzothiadiazine-7-sulfonamide,1,1-dioxide (Figure 1).

The TLC experiments were performed on 10 cm × 10 cm cellulose (Art. 105552, Merck, Germany) layers. The plates were spotted with 2 μL aliquots of freshly prepared aqueous solution of lisinopril and ethanolic solutions of enalapril, quinapril, cilazapril and HCTZ (2 mg/mL), and were developed via the ascending technique.

After the development, the detection was performed by exposing the plates to iodine vapour. All investigations were performed at room temperature (22 ± 2ºC).

The mobile phases used are presented in Table 1.

**Table 1:** hR_F_-values of the investigated substances

**Substance**	**Water-methanol**					**Water-acetone**					**Water-ethanol**				
	**a**10	15	20	25	30	10	15	20	25	30	10	15	20	25	30
**1**	78	80	83	86	88	78	84	86	89	93	81	85	90	92	93
**2**	53	58	65	68	71	55	60	66	70	73	65	70	74	79	82
**3**	85	88	91	92	93	86	90	92	94	96	91	93	95	96	97
**4**	60	68	75	80	85	66	70	73	77	80	73	76	79	83	88
**HCTZ**	80	83	85	90	92	83	85	88	92	95	85	87	90	93	95

^a ^Vol% of organic modifier in mobile phases

## Conclusions

The R_F_ -values were calculated according to the relationship:

R_F _= Distance of spot from origin / Distance of mobile phase front from origin

Here, R_M_-values were calculated for each solute in each mobile phase according to the Bate-Smith and Westall equation (29):

$R_{M}=\log(\frac{1}{R_{F}}-1)$          (Equation 1)

The log *p *values (*KOWWIN) *of the examined compounds were calculated using software (30).

## Results and Discussion

The systematic investigation of four ACE inhibitors and HCTZ was performed using RP-TLC method on cellulose layers by means of three water-organic modifier binary solvent systems. In an aim to establish the reversed-phase TLC on cellulose, the mobile phase had to be more polar than the cellulose. For that purpose, the binary solvent systems used had a relatively low content of organic modifier (methanol, acetone and ethanol) 10 to 30% (with 5% intervals).

The results (Table 1) show that the increase in the concentration of organic modifier in mobile phase leads to the increase of hR_F _(R_F_ × 100) values, *i.e*. to a decrease of the retention of the investigated substances. For the different mobile phases with the same water content, the retention is decreased from methanol to ethanol as the solvent polarity is decreased (31). Irrespectively to the structural differences between the investigated substances, the same retention order of compounds was established for all used mobile phases: R_F_ (3) > R_F_ (5) > R_F_ (1) > R_F_ (4) > R_F_ (2).

The retention behavior of biological active substances investigated in RP-TLC can be presented as the relationship between the R_M_-values and the content of organic modifier in mobile phase through the linear Equation (16):

R_M_ = R^0^_M_ + mC          (Equation 2)

The value of the intercept, R^0^_M_, represents the lipophilicity of the examined substance and the value of the slope, m, corresponds to the specific hydrophobic surface area of this substance, while C represents the volume fraction of the organic modifier in mobile phase.

By the analogy through the hydrophobicity parameter of φ_0_ (6, 7), previously defined for the HPLC method as the concentration of the organic modifier in the mobile phase for which the distribution of the analyzed substance between the mobile and stationary phase was equal (1:1) another hydrophobicity parameter, C_0_, can be calculated. The hydrophobicity parameter, C_0_, represents the volume fraction of the organic modifier in mobile phase where R_M_ = 0 (11-12) and it can be calculated as C_0_ = - R^0^_M_ / m.

The chromatographically obtained hydrophobicity parameters including slope (m), intercept (R^0^_M_) and C_0_ for each mobile phase are presented in Table 2.

**Table 2:** Regression analysis and hydrophobicity parameters of investigated compounds

	**R**_M_^0^	**- m**	**- r**	**C**_0_
**Substance**	**Water-methanol** ^*^			
**1**	- 0.372 ± 0.019	1.635 ± 0.001	0.995	- 0.227
**2**	0.109 ± 0.032	1.721 ± 0.001	0.988	0.063
**3**	- 0.587 ± 0.042	1.871 ± 0.002	0.983	- 0.313
**4**	0.104 ± 0.010	2.858 ± 0.001	0.999	0.036
**HCTZ**	- 0.339 ± 0.054	2.366 ± 0.023	0.982	- 0.143
**Substance**	Water-acetone^*^			
**1**	- 0.284 ± 0.057	2.671 ± 0.002	0.985	- 0.106
**2**	0.082 ± 0.020	1.763 ± 0.001	0.995	0.047
**3**	- 0.506 ± 0.031	2.849 ± 0.001	0.996	- 0.177
**4**	- 0.129 ± 0.009	1.569 ± 0.001	0.999	- 0.082
**HCTZ**	- 0.334 ± 0.081	2.975 ± 0.004	0.976	- 0.112
**Substance**	Water-ethanol^*^			
**1**	- 0.386 ± 0.057	2.589 ± 0.002	0.984	- 0.149
**2**	- 0.070 ± 0.013	1.974 ± 0.001	0.998	- 0.036
**3**	- 0.753 ± 0.017	2.533 ± 0.001	0.998	- 0.297
**4**	- 0.191 ± 0.054	2.109 ± 0.002	0.978	- 0.090
**HCTZ**	- 0.448 ± 0.046	2.697 ± 0.002	0.990	- 0.166

^*^ The volume range of organic modifiers in mobile phases was 10-30%.

It has been shown in the literature that there is usually a linear correlation between the intercept, R^0^_M_, and slope, m. The linear correlations for intercept and slope values were established for:

Water-acetone, R^0^_M_ = (0.4384 ± 0.2659) + (0.2843 ± 0.1091) m, with r = 0.8327 and SD = 0.1421

as well as:

Water-ethanol, R^0^_M_ = (1.1358 ± 0.7277) + (0.6323 ± 0.3035) m, with r = 0.7689 and SD = 0.1935

The good correlations reflect the suitability of the systems examined for estimating the lipophilicity of the compounds and can indicate that the investigated substances, ACE inhibitors and HCTZ, could be considered as compounds belonging to the same group under the described conditions. Only for water-methanol solvent system, the correlation coefficient was significantly lower (r = 0.3836).

As shown in Table 2, the hydrophobicity parameters, R^0^_M_ and C_0_ obtained in these investigations were mostly increased with increase of compounds lipophilicity (log *P*_1 _= 2.45, log *P*_2_ = 3.72, log *P*_3_ = - 0.94, log *P*_4 _= 2.27, log *P*_HCTZ_ = - 0.10) (30). By comparison hydrophobicity parameters (R^0^_M_ and C_0_) with retention data (Table 1) of investigated ACE inhibitors and HCTZ, the retention order obtained on cellulose layers using water-acetone and water-ethanol correlates completely with both hydrophobicity parameters.

**Figure 2:**
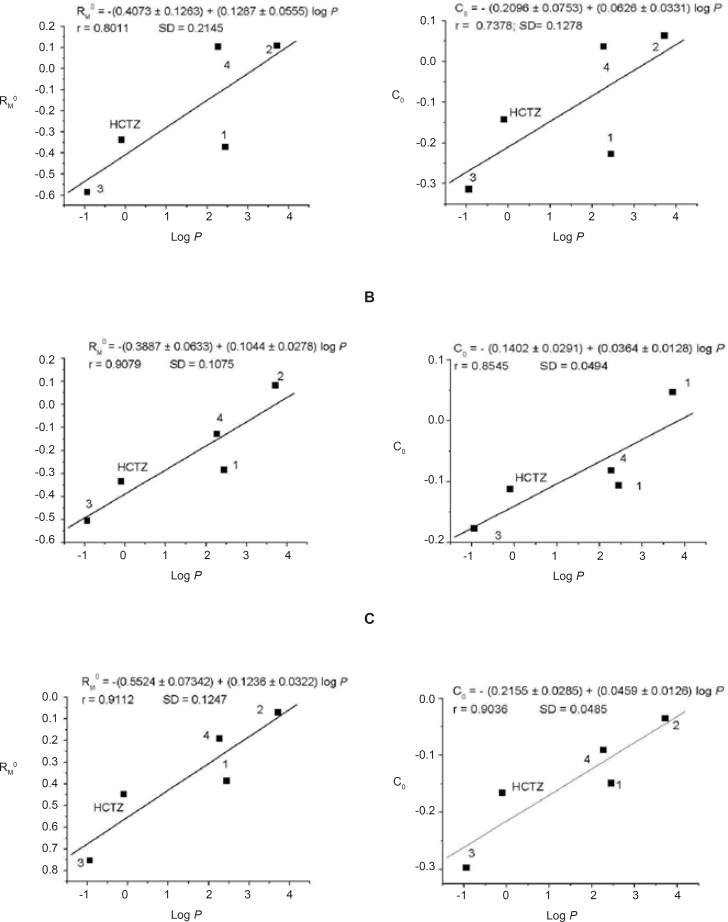
Correlation between the hydrophobicity parameters R_M_^0^ and C_0_ and calculated log *p *values of investigated substances for different mobile phases: water-methanol (A), water-acetone (B) and water-ethanol (C). The volume range of organic modifiers in mobile phases was 10-30%. The numbers denote examined substances

In order to evaluate the possibility of the applying cellulose in RP-TLC for the determination of selected ACE inhibitors and HCTZ lipophilicity, the chromatographically established hydrophobicity parameters, R^0^_M_ and C_0_, were correlated with calculated *log p *values *(KOWWIN) *(30). Calculated log *p *values fully correlate with experimentally determinated octanol-water partition coefficient, log *p *values (32). The established relations are shown at Figure 2 and the satisfactory correlation was observed in all cases.

The presented results demonstrate that the most lipophilic compound among those investigated was quinapril. On the other hand, lisinopril and diuretic HCTZ, were the most hydrophilic due to their amino-acid moiety and sulfonamide group, respectively.

In addition, the correlations between the chromatographically established hydrophobicity parameters, R^0^_M_ and C_0_, with calculated log *p *values obtained through the RP-TLC on cellulose were compared with those previously obtained on RP-18 silica gel using the same solvent systems (27). For RP-18 silica gel, these correlations were obtained with recalculated log *p *values (30) with the addition of HCTZ which was not previously examined. The correlation coefficients established on cellulose were in the range of 0.7378-0.9112 (the p-values were 0.1547-0.0313; obtained using Origin 7), while those obtained on RP-18 silica gel were 0.8545-0.9706 (the p-values were 0.0651-0.0060). Our results indicate that the high correlations (33) were obtained on both sorbents.

Furthermore, the hydrophobicity parameters, C_0_, obtained with cellulose support were correlated with the corresponding C_0_ parameters previously obtained on RP-18 silica gel (with the addition of hydrochlorothiazide C_0_ parameter) using the same solvent systems. Very good correlations were observed and the correlation coefficients were in the range of 0.8084-0.9137 (the p-values were 0.0977-0.0300). The obtained data could indicate that the cellulose support is sufficiently reliable for chromatographic lipophilicity investigations of ACE inhibitors and HCTZ.

By considering the suitability of the applied mobile phases, it can be seen that water-acetone and water-ethanol shows better correlations than water-methanol and they are more suitable for the lipophilicity determinations of examined ACE inhibitors and HCTZ.

These findings discussed above, make it obvious that cellulose as an economic, inexpensive and easily available sorbent can be used as a successful alternate to RP-18 silica gel in RP-TLC investigations of ACE inhibitors and HCTZ lipophilicity.

## Conclusion

Defining an appropriate method and also conditions for rapid, simple and inexpensive determination of lipophilicity is essential for the investigation of biological active substances.

This study shows satisfactory correlations between parameters, R^0^_M_ and C_0_, as the measures of hydrophobicity, with the calculated log *p *values, showing that cellulose can be efficiently applied in the RP-TLC investigation of lipophilicity of the selected ACE inhibitors and HCTZ. Very good correlation (r = 0.91; water-ethanol solvent system) between the chromatographically obtained hydrophobicity parameters and calculated log *p *values confirmed the selection of ACE inhibitors since lisinopril and quinapril were on the opposite sites of linear relationship. The principal advantage of cellulose compared to the other sorbents commonly used in RP-TLC (RP-18 silica gel) is that cellulose is an easily accessible sorbent. Moreover, lower organic solvent content in the eluent needed to achieve the consistent retention follows the principles of green chemistry.
